# Air pollutants and outpatient visits for cardiovascular disease in a severe haze-fog city: Shijiazhuang, China

**DOI:** 10.1186/s12889-019-7690-4

**Published:** 2019-10-24

**Authors:** Fengzhu Tan, Weijie Wang, Sufen Qi, Haidong Kan, Xinpei Yu, Yi Liu, Duanyang Wu, Bin Xu, Fan Meng, Sicen Liu

**Affiliations:** 1 Department of Environmental and Occupational Health, School of Public Health, Hebei Medical University, 361 Zhongshan East Road, Shijiazhuang, 050017 Hebei China; 2 Departments of Toxicology, School of Public Health, Hebei Medical University, Shijiazhuang, China; 3 Department of Outpatient Medical Record, The Second Hospital of Hebei Medical University, Shijiazhuang, China; 4 School of Public Health, Key Lab of Public Health Safety (Ministry of Education), Fudan University, Shanghai, China; 5 Hebei Society for Environmental Sciences, Shijiazhuang, China; 6 Department of of Toxicology, School of Public Health, Hebei Medical University, Shijiazhuang, China

**Keywords:** Air pollution, Cardiovascular disease, Outpatient visits, Particulate matter, Sulphur dioxide, Nitrogen dioxide, Carbon monoxide

## Abstract

**Background:**

Many studies have reported the impact of air pollution on cardiovascular disease (CVD), but few of these studies were conducted in severe haze-fog areas. The present study focuses on the impact of different air pollutant concentrations on daily CVD outpatient visits in a severe haze-fog city.

**Methods:**

Data regarding daily air pollutants and outpatient visits for CVD in 2013 were collected, and the association between six pollutants and CVD outpatient visits was explored using the least squares mean (LSmeans) and logistic regression. Adjustments were made for days of the week, months, air temperature and relative humidity.

**Results:**

The daily CVD outpatient visits for particulate matter (PM_10_ and PM_2.5_), sulphur dioxide (SO_2_), nitrogen dioxide (NO_2_), carbon monoxide (CO), and ozone (O_3_) in the 90th-quantile group were increased by 30.01, 29.42, 17.68, 14.98, 29.34%, and − 19.87%, respectively, compared to those in the <10th-quantile group. Odds ratios (ORs) and 95% confidence intervals (CIs) for the increase in daily CVD outpatient visits in PM_10_ 300- and 500-μg/m^3^, PM_2.5_ 100- and 300-μg/m^3^ and CO 3-mg/m^3^ groups were 2.538 (1.070–6.020), 7.781 (1.681–36.024), 3.298 (1.559–6.976), 8.72 (1.523–49.934), and 5.808 (1.016–33.217), respectively, and their corresponding attributable risk percentages (AR%) were 60.6, 87.15, 69.68, 88.53 and 82.78%, respectively. The strongest associations for PM_10_, PM_2.5_ and CO were found only in lag 0 and lag 1. The ORs for the increase in CVD outpatient visits per increase in different units of the six pollutants were also analysed.

**Conclusions:**

All five air pollutants except O_3_ were positively associated with the increase in daily CVD outpatient visits in lag 0. The high concentrations of PM_10_, PM_2.5_ and CO heightened not only the percentage but also the risk of increased daily CVD outpatient visits. PM_10_, PM_2.5_ and CO may be the main factors of CVD outpatient visits.

## Background

Over the past several decades, air pollution has been thought to have adverse effects on public health. Many epidemiological studies have revealed that primary ambient air pollutants, including PM_2·5_, PM_10_, NO_2_, SO_2_, O_3_ and CO, increase the risk of hospital admission and mortality for CVD [1–9]. Exposure to air pollutants, except for O_3,_ was strongly associated with acute cardiovascular events such as myocardial infarction, heart failure and arrhythmia [10, 11]. Only one study has shown that acute controlled exposure to air pollutants did not increase the short-term risk of arrhythmia in participants [12]. Analytical methods as time series analysis, generalized additive models and case-crossover were used to estimate the adverse effects of air pollutants on the hospitalization and mortality of CVD in some studies in the United States, Canada, the United Kingdom and the moderately polluted areas of China (e.g., Shanghai: the average PM_10_ and PM_2.5_ were, respectively, 81.7 μg/m^3^ and 38.6 μg/m^3^) [3, 4, 13, 14]. PM_2·5_ in developed countries is relatively low (median 15 μg/m^3^), but PM_2·5_ in some cities in developing countries is likely to be up to 10-fold higher than the US National Ambient Air Quality Standards. New Delhi in India and Beijing in China have daily PM_2·5_ concentrations of 100–300 μg/m^3^. More high-quality studies are urgently needed to establish the effect of air pollution on heart failure outcomes in developing countries [10].

In 2013, severe haze-fog events occurred frequently in northern China and were more concentrated in some cities in Hebei Province around Beijing. However, the acute impact of air pollutants on CVD in these severe haze-fog areas has rarely been reported. In addition, it seems unsuitable to assess the impact of PM_2.5_ on CVD in these severe haze-fog areas using the methods (e.g., only evaluating the impact of PM_2.5_ per 10-μg/m^3^ increase) used in developed countries or in lightly or mildly polluted areas because the PM_2.5_ daily average concentration (sometimes up to 500 μg/m^3^) or annual average concentration in developing countries was much higher than in developed countries. Therefore, it is necessary to identify new methods to explore or explain the relationship between short-term exposure to air pollutants and outpatient visits or hospitalization for CVD in these severe haze-fog areas.

Based on the above, we hypothesize that the relationship between daily CVD outpatient visits and air pollutants in the severe haze-fog areas in developing countries should differ from this relationship in moderately or slightly polluted areas in developed countries. Therefore, we focused on the concentration-impact relationship between the different daily concentrations of air pollutants and the daily number of CVD outpatient visits (i.e., evaluating the impact of air pollutants by calculating ORs of different concentrations, not merely by calculating the ORs of each 10-μg/m^3^ increase). Our results will provide an early warning reference for managers in hospitals and in the Centers for Disease Control and Prevention during the occurrence of persistent severe haze-fog.

## Methods

### Study design and data collection

Shijiazhuang belongs to the region of Beijing-Tianjin-Hebei (BTH) and was one of 10 most heavily polluted cities in 2013 in China, where the median daily concentrations for PM_10_ and PM_2.5_ were up to 287 μg/m^3^ and 123 μg/m^3^, respectively. The data on average concentrations of six daily air pollutants (PM_10_, PM_2.5_, SO_2_, NO_2_, O_3_ and CO) were from the national environmental monitoring points in Shijiazhuang. Daily meteorological data (air temperature and relative humidity) were provided by Beijing’s National Meteorological Center. The data for daily outpatient visits (including emergency room patients) for CVD were collected from the four branch hospitals of two affiliated hospitals of Hebei Medical University, where all electronic medical records were classified according to the International Classification of Diseases (10th Revision) and each patient had a complete medical record (e.g., name, sex, region for patients; department for diagnosis, disease type, disease name, and ICD code). We chose the outpatients in ICD-10 codes I00–I99, including CVD, such as angina, arrhythmia, heart failure, and myocardial infarction. All CVD outpatients were identified by two data collectors. We only collected outpatient data that was not related to personal privacy (i.e., de-identification data that did not include personal name, gender, ethnicity, body weight, etc.) and the study protocol was approval by Medical Ethics Committee in Hebei Medical University. The network technology has been widely put into use in 2013, and ICD coding records for outpatient visits in the hospitals were more functional than ever, which helped us to correctly choose the CVD outpatients. All data were collected from January 1 to December 31, 2013.

### Variables and statistical analysis

We obtained the least squares mean (LSmeans) of daily CVD outpatient visits in every decile group of each air pollutant using a general linear model (GLM of SAS) to better understand and explore the relationship between the daily concentration of six air pollutants (independent variables) and the daily amount of CVD outpatient visits (dependent variables). The difference between the LSmeans of CVD in the 10 quantile groups of each air pollutant was also tested. Based on the LSmeans, we calculated the increased percentage of daily CVD outpatient visits in 9 quantile groups of each air pollutant decile compared with the < 10th-quantile group.

Non-conditional logistic regression was used to calculate ORs and 95% CIs to further evaluate the risk of the increase of CVD outpatient visits in different concentrations and lag days for each pollutant. Based on the above LSmeans results of CVD outpatient visits, the concentration of each air pollutant was re-grouped. The lowest concentration of each air pollutant was used as a reference level for calculating OR, for which concentrations for PM_10_, PM_2.5_, CO, SO_2_ and NO_2_ were < 200 μg/m^3^, < 100 μg/m^3^, < 1 mg/m^3^, < 50 μg/m^3^ and < 40 μg/m^3^, respectively. If the number of daily CVD outpatient visits was > 285 (i.e., more than average of one year), the amount of daily CVD outpatient visits was considered to have increased (i.e., more than average), which was considered to be a threshold for the increase in daily CVD outpatient visits); in contrast, if the number of daily CVD outpatient visits was ≤285, it was considered to show no increase (i.e., considered to be the usual number of daily CVD outpatient visits). Moreover, we first analysed LSmeans of daily CVD outpatient visits at the same concentrations before calculating the above ORs, which would allow LSmeans and ORs to support or confirm each other.

We also analysed the following: the decile concentration values of each pollutant (supplemental files for the above content); the ORs and 95% CIs for CVD outpatient visits per increase in different unit of each pollutant; and the Pearson partial correlation coefficient between CVD outpatient visits and air pollutants on different lag days.

Regarding the confounding factors, we considered the results of preliminary analysis (the amount of CVD outpatient visits decreased along with air temperature increasing, but air humidity had no such impact) in our data on the one hand; on the other hand, referencing the results of other similar studies, days of the week, months, daily air temperature and relative humidity (the air temperature and humidity were also divided into ten groups by decile and then adjusted) were identified as confounding factors. These confounders were adjusted in all the above analyses (LSmeans, ORs, and Pearson partial correlation coefficient). However, the flu was not adjusted for because the proportion of flu outpatient visits in northern China in 2013 did not exceed the highest level in the 5 years before 2013 [15].

In addition, descriptive analysis, cluster analysis and other basic analyses were also performed prior to the above analyses to understand the characteristics of air pollutants or CVD outpatient visits and the confounding factors to be adjusted. These analyses showed that O_3_ did not belong to the same category of variables as PM_10_, PM_2.5_, SO_2_, NO_2_, and CO and was negatively related to CVD outpatient visits; thus, the O_3_ data from 183 days of the warm period (16 April to 15 October) were analysed as well.

A flow diagram for statistical analyses was provided in Additional file 1: Figure S1. All statistical analyses were performed using SAS software (SAS Institute Inc. Contract Site Number: 553024). A *P* value < 0.05 was considered statistically significant.

## Results

### LSmeans of daily CVD outpatient visits

**Fig. 1:**
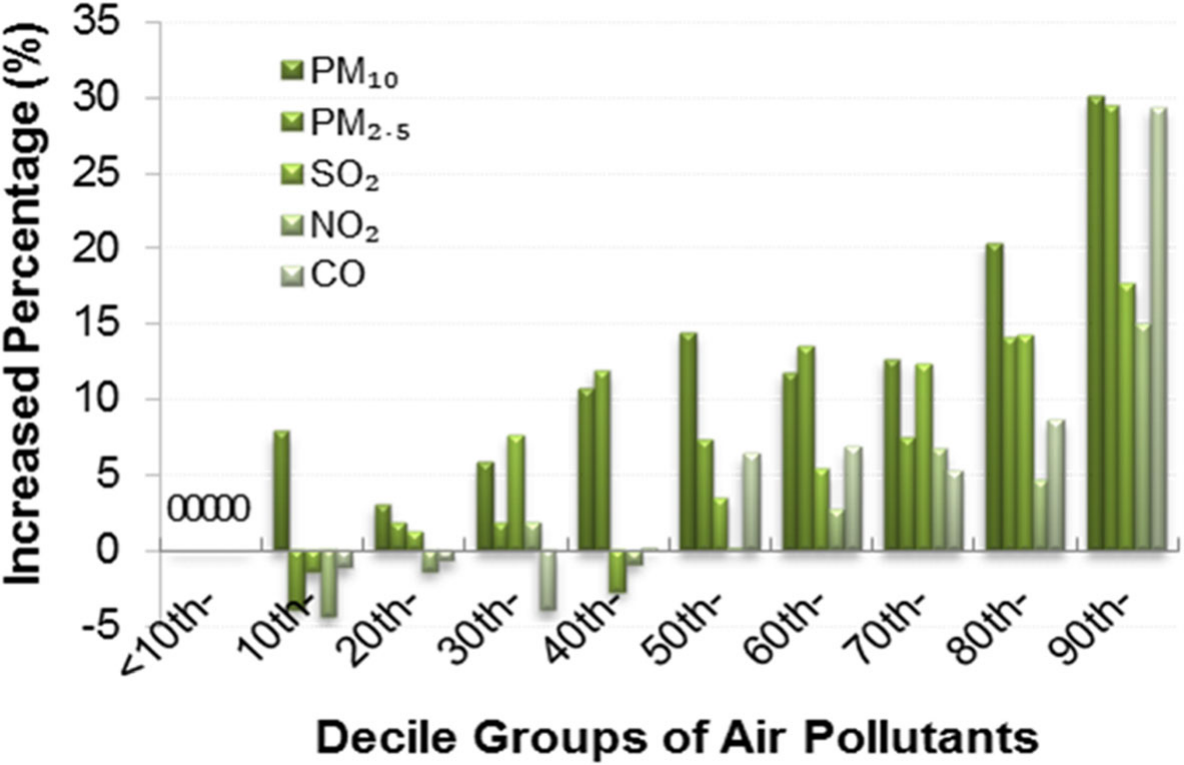
The increased percentage of daily CVD outpatient visits in nine quantile groups for different air pollutants in lag 0 compared with the <10th- group

Table 1 provides the LSmeans of daily CVD outpatient visits in the different quantile groups of air pollutants in Lag 0. The LSmeans of the PM_10_ 50th group, the PM_2.5_ 40th group, the SO_2_ and NO_2_ 70th group, and the CO 80th group began to be higher than those of other groups (all *P* < 0.05). However, O_3_ showed a negative relationship with daily CVD outpatient visits, which was different from other air pollutants; daily CVD outpatient visits in the 80th group was significantly higher than those in the 40th group only in the warm period (183 days, *P* < 0.05). In addition, the 90th-quantile concentration values for PM_10_, PM_2.5_, SO_2_, NO_2_, CO, O_3_ (365 days) and O_3_ (183 days) were 4.0, 7.1, 9.1, 2.8, 6.1, 14.4 and 3.6 times the 10th-quantile concentration values, respectively (see Additional file 1: Table S1). Among them, the highest daily average concentrations of PM_10_ (990 μg/m^3^) and PM_2.5_ (751 μg/m^3^) were 6.6 times and 21.5 times the US air quality standard (PM_10_ = 150 μg/m^3^, PM_2.5_ = 35 μg/m^3^) and 6.6 times and 10.0 times the China air quality standard (PM_10_ = 150 μg/m^3^, PM_2.5_ = 75 μg/m^3^). The highest daily average concentration of PM_10_ (990 μg/m^3^) was 19.8 times the EU air quality standard (PM_10_ = 50 μg/m^3^) and one-quarter of the particulates concentration (4460 μg/m^3^) in the London smog incident of 1952. Figure 1 shows the increased percentage of daily CVD outpatient visits in 9 quantile groups of each air pollutant in lag 0 compared with the <10th-quantile group. The increased percentages in the 90th-quantile group for PM_10_, PM_2.5_, SO_2_, NO_2_ and CO were 30.01, 29.42, 17.68, 14.98 and 29.34%, respectively. In short, the increase in CVD for PM_10_ and PM_2.5_ was higher than that in SO_2_ and NO_2_; the increase of CVD for CO was obvious only in the 90th-quantile group. Table 1 and Fig. 1 revealed a different relationship between PM_10_ or PM_2.5_ concentrations and CVD outpatient visit amount.

**Table 1:** Daily outpatient visits for cardiovascular disease in different decile groups for air pollutants in lag 0

Decile groups	Daily outpatient visits^a^						
	PM_10_	PM_2.5_	SO_2_	NO_2_	CO	O_3_	O_3_^c^
<10th-	244 ± 10	252 ± 11	258 ± 13	266 ± 10	259 ± 13	329 ± 15^b^	265 ± 13
10th-	263 ± 9	242 ± 9	254 ± 10	254 ± 9	256 ± 9	307 ± 12^b^	269 ± 11
20th-	252 ± 9	256 ± 9	261 ± 10	263 ± 10	257 ± 9	284 ± 9^b^	247 ± 11
30th-	259 ± 9	256 ± 9	277 ± 9	271 ± 8	248 ± 11	266 ± 10	246 ± 11
40th-	271 ± 9	282 ± 9^b^	250 ± 9	264 ± 10	259 ± 8	265 ± 9	241 ± 10
50th-	280 ± 8^b^	270 ± 9^b^	267 ± 10	267 ± 9	275 ± 9	266 ± 9	259 ± 11
60th-	273 ± 9^b^	286 ± 9^b^	271 ± 10	273 ± 9	277 ± 8	253 ± 10	267 ± 11
70th-	275 ± 9^b^	271 ± 9^b^	289 ± 9^b^	284 ± 9^b^	272 ± 8	242 ± 10	254 ± 10
80th-	294 ± 9^b^	287 ± 9^b^	294 ± 11^b^	278 ± 10	280 ± 11^b^	261 ± 11	274 ± 11^b^
90th-	318 ± 10^b^	326 ± 11^b^	303 ± 13^b^	306 ± 11^b^	336 ± 13^b^	264 ± 11	270 ± 12

^a^Numbers w ere LSmeans±SE, adjusted for days of the w eek, months, air temperature and relative humidity

^b^LSmeans in these groups w ere signif icantly higher than those in other groups (*P* < 0.05)

^c^Results f rom the w arm period (16 April to 15 October, 183 days)

### Impact of PM_10_ on CVD outpatient visits

**Fig. 2:**
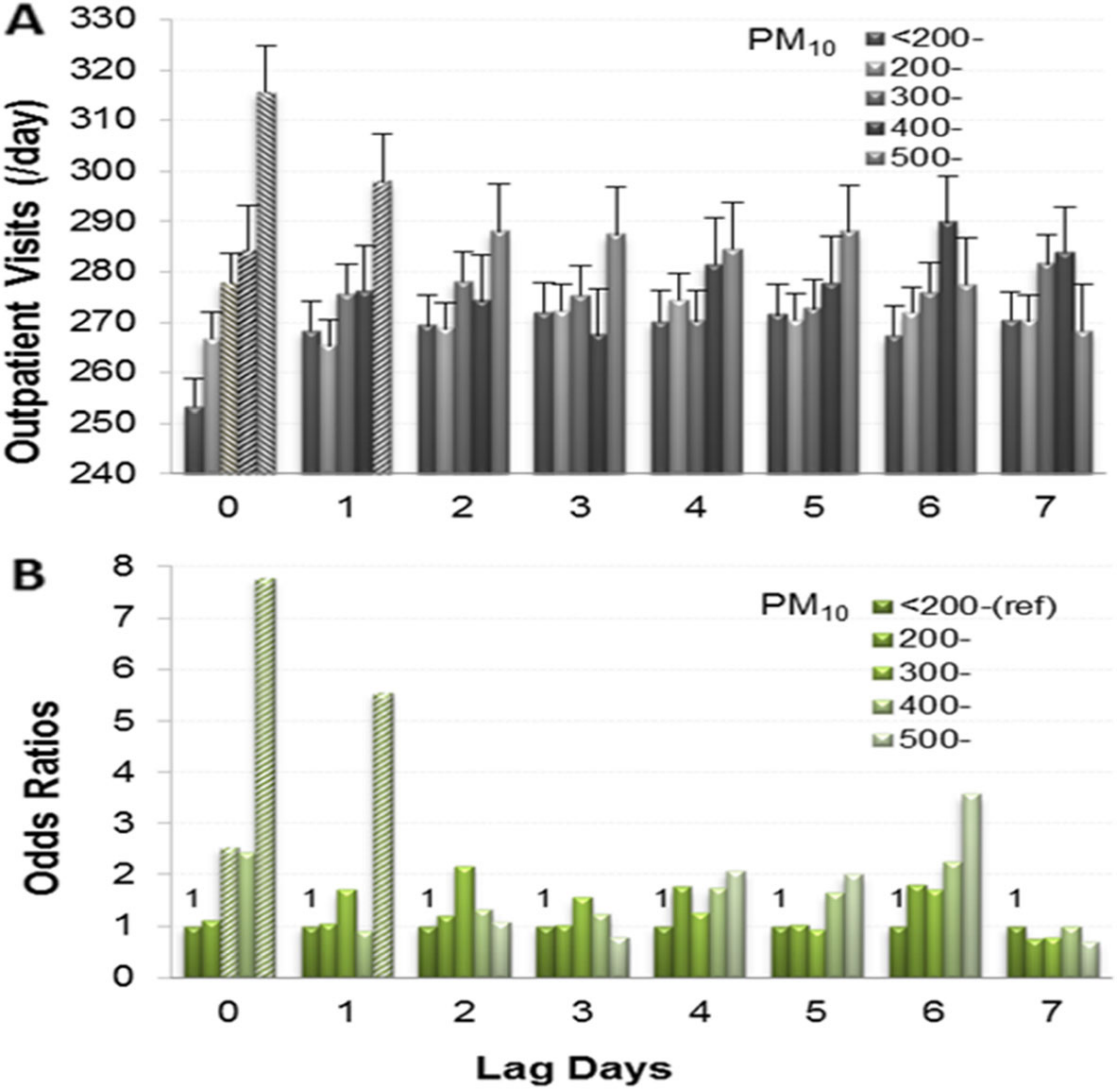
Relationship between PM_10_ concentration (μg/m^3^) and CVD outpatient visits on different lag days. **a**: Daily CVD outpatient visits (LSmeans±SE). **b**: Odds ratios for the increase in daily CVD outpatient visits. A and B were adjusted for days of the week, months, air temperature and relative humidity. Italics indicate significant differences (*P* < 0.05)

Figure 2 provides the impact of PM_10_ at different concentrations (μg/m^3^) and lag days on CVD outpatient visits. In panel A, the LSmeans of lag 0 and 1 in the 500-μg/m^3^ group were higher than those in the other four groups; the LSmeans of lag 0 in the 400-μg/m^3^ and 300-μg/m^3^ groups were higher than those in the < 200-μg/m^3^ group (all *P* < 0.05). In panel B, the ORs (95% CIs) of lag 0 in the 200-μg/m^3^, 300-μg/m^3^, 400-μg/m^3^ and 500-μg/m^3^ groups were 1.113 (0.523–2.367), 2.538 (1.07–6.02), 2.434 (0.709–8.364) and 7.781 (1.681–36.024), respectively, and their AR% were 10.15, 60.60, 58.92 and 87.15%, respectively. The OR (95% CI) of lag 1 in the 500-μg/m^3^ group was 5.56 (1.293–23.899), and its AR% was 82.01%. Whether LSmeans or ORs, the impact of PM_10_ in the 500-μg/m^3^ group on CVD was obvious in lags 0 and 1.

### Impact of PM_2.5_ on CVD outpatient visits

**Fig. 3:**
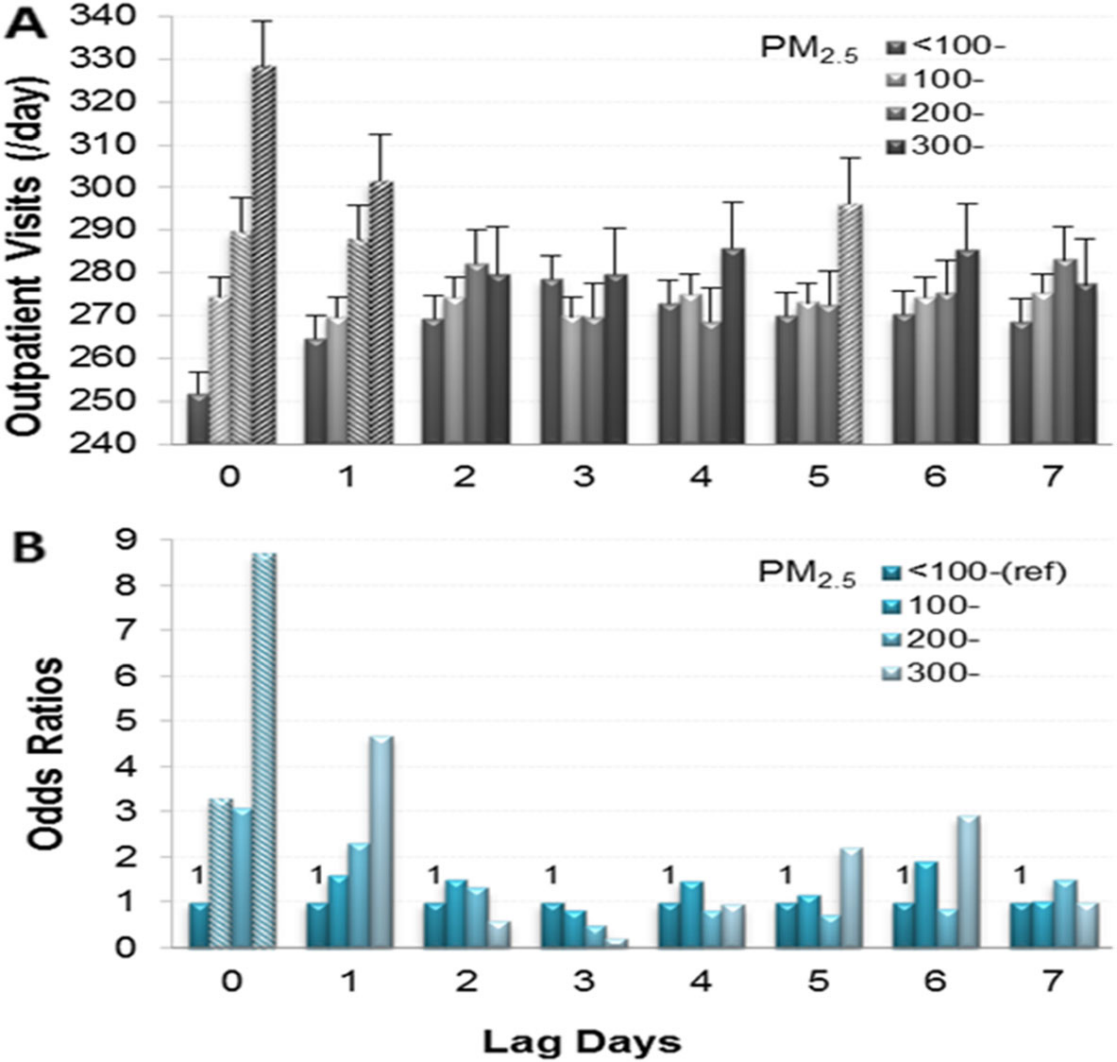
Relationship between PM_2.5_ concentration (μg/m^3^) and CVD outpatient visits on different lag days. **a**: Daily CVD outpatient visits (LSmeans±SE). B: Odds ratios for the increase in daily CVD outpatient visits. **a** and **b** were adjusted for days of the week, months, air temperature and relative humidity. Italics indicate significant differences (*P* < 0.05)

Figure 3 provides the impact of PM_2.5_ at different concentrations (μg/m^3^) and lag days on CVD outpatient visits. In panel A, the LSmeans of lag 0 in the 300-μg/m^3^ group were higher than those in the other three groups; the LSmeans of lag 0 in the 200-μg/m^3^ and 100-μg/m^3^ groups were higher than those in the < 100-μg/m^3^ group. The LSmeans of lag 1 in the 300-μg/m^3^ and 200-μg/m^3^ groups were higher than those in the 100-μg/m^3^ and < 100-μg/m^3^ groups (all *P* < 0.05). In panel B, the ORs (95% CIs) of lag 0 in the 100-μg/m^3^, 200-μg/m^3^ and 300-μg/m^3^ groups were 3.298 (1.559–6.976), 3.094 (0.939–10.196) and 8.72 (1.523–49.934), respectively, and their AR% were 69.68, 67.68 and 88.53%, respectively. Although the ORs of lag 1 were not significant, the ORs showed an increasing trend. The LSmeans of lag 5 in the 300-μg/m^3^ group were significantly higher than those in the 200-μg/m^3^ group, but the OR was not significant. Whether LSmeans or ORs, the impact of PM_2.5_ in the 300-μg/m^3^ group on CVD was obvious only in lag 0.

### Impact of CO on CVD outpatient visits

**Fig. 4:**
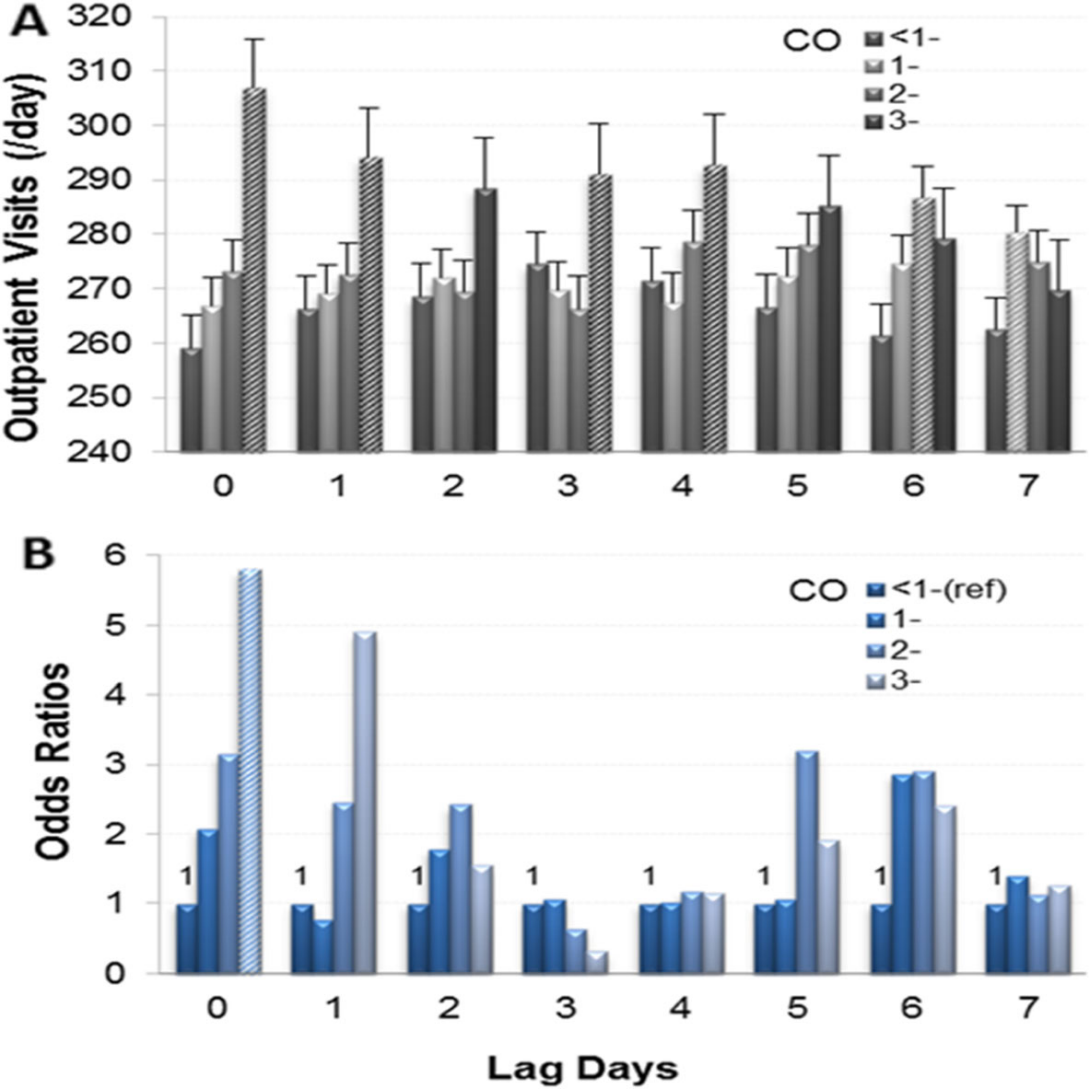
Relationship between CO concentration (mg/m^3^) and CVD outpatient visits on different lag days. **a**: Daily CVD outpatient visits (LSmeans±SE). **b**: Odds ratios for the increase in daily CVD outpatient visits. **a** and **b** were adjusted for days of the week, months, air temperature and relative humidity. Italics indicate significant differences (*P* < 0.05)

Figure 4 provides the impact of CO at different concentrations (mg/m^3^) and lag days on CVD outpatient visits. In panel A, the LSmeans of lag 0, 1, 3 and 4 in the 3-mg/m^3^ group were higher than those in the other three groups; the LSmeans of lag 6 in the 2-mg/m^3^ group and the LSmeans of lag 7 in the 1-mg/m^3^ group were higher than those in the < 1-mg/m^3^ group (all *P* < 0.05). In panel B, the ORs (95% CI) of lag 0 in the 1-mg/m^3^, 2-mg/m^3^ and 3-mg/m^3^ groups were 2.076 (0.98–4.396), 3.148 (0.795–12.473) and 5.808 (1.016–33.217), respectively, and their AR% were 51.83%, 68.23 and 82.78%, respectively. Although some LSmeans on other lag days except for lag 0 were significantly higher, the ORs were not significant. Whether LSmeans or ORs, the impact of CO in the 3-mg/m^3^ group on CVD was obvious in lag 0.

### Impact of SO_2_ and NO_2_ on CVD outpatient visits

However, the increase in daily CVD outpatient visits at different concentrations (μg/m^3^) of SO_2_ (< 50-μg/m^3^, 50-μg/m^3^, 100-μg/m^3^ and 150-μg/m^3^ groups) or NO_2_ (< 40-μg/m^3^, 40-μg/m^3^, 70-μg/m^3^ and 100-μg/m^3^ groups) were not as obvious as those of PM_10_, PM_2.5_ and CO. Although the LSmeans in some groups were higher than those in other groups (between the 150-μg/m^3^ and 50-μg/m^3^ groups for SO_2_; between the 100-μg/m^3^ and 40-μg/m^3^ groups for NO_2_), all ORs for the increase in outpatient visits were not significant (not shown).

### Other supplementary analyses

Additional multiple logistic stepwise regression analyses also showed that PM_2.5_ (OR = 2.268, CI: 1.142–4.504) and CO (OR = 2.687, CI: 1.114–6.479) were the main risk factors for the increase in daily CVD outpatient visits (not shown). Moreover, Additional file 1: Table S2 also provides the ORs of daily CVD outpatient visits in lag 0 per increase in different units of each pollutant. The results showed that PM_2.5_, PM_10_ and CO increased the risk of the increase in CVD outpatient visits. PM_2.5_, PM_10_ and CO can be considered the main risk factors according to the results of the ORs and standardized estimates. However, the ORs from SO_2_, NO_2_, and O_3_ (in 183 days of the warm period) were not significant, and the standardized estimate from O_3_ (in 365 days) was unexpectedly negative. The trend of results in Additional file 1: Table S2 was consistent with the LSmeans (Table 1), the increased percentage (Fig. 1) and the ORs (B of Fig. 2, 3, 4), although those numerical values were different. Additional file 1: Table S3 provides the Pearson partial correlation coefficient between daily CVD outpatient visits and air pollutants on different lag days; the trend of those coefficients was also consistent with results from Table 1, Fig. 1 and Additional file 1: Table S2, and most of the coefficients for O_3_ (in 365 days) were also negative.

## Discussion

Several epidemiological (observational) studies have shown that hospitalization or death due to acute heart failure is associated with increased concentrations of PM_10_, PM_2.5_, CO, SO_2_, and NO_2_ [2–6, 8, 9]. However, few published articles have used LSmeans and logistic regression to explore the relationship between acute exposure to air pollutants and increased CVD outpatient visits in a severe haze-fog city. In this study, we evaluated the increased percentage and risk (OR) of the increase in daily CVD outpatient visits for different concentrations of air pollutants using LSmeans and logistic regression. Daily CVD outpatient visits increased by approximately 30% in the 90th-quantile group (PM_10_ ≥ 535 μg/m^3^, PM_2.5_ ≥ 304 μg/m^3^, CO ≥ 4.3 mg/m^3^), which was consistent with the television reports when severe haze-fog occurred. Our results, which were expressed using LSmeans and the increased percentages, should be easier to understand for the general population.

### Impact of Particulate Matter

Many studies have shown that short-term exposure to PM_10_ or PM_2.5_ was significantly associated with an increase in hospital admissions for CVD [3–7, 10, 11, 13, 14, 16–23]. PM_10_ and PM_2.5_ increased the risk of hospitalization for heart failure (PM_10_ 1.63% per 10-μg/m^3^ increase, CI: 1.20–2.07; PM_2.5_ 2.12% per 10-μg/m^3^ increase, CI: 1.42–2.82). The strongest association was often observed on the day of exposure [4, 10, 21]. PM_2.5_ had a stronger impact on heart failure than other cardiovascular diseases, with 3.1% of heart failure admissions attributable to short-term PM_2.5_ exposure over background levels of 5 μg/m^3^. Older adults were significantly more susceptible to heart failure than younger adults after short-term PM_2.5_ exposure [17]. In Beijing, China, after adjustments were made for the temperature and relative humidity, the ORs for CVD in hospital emergency room visits for 10-μg/m^3^ increases in PM_2.5_ were 1.005 (95% CI: 1.001–1.009) [7]. In Shanghai, China, a 10-μg/m^3^ increase in PM_10_ and PM_2.5_ in 2 days resulted in an increase of 0.23% (CI: 0.12–0.34) and 0.74% (CI: 0.44–1.04), respectively, in the morbidity of coronary heart disease [14]. In our study, PM_10_ (the 90th-quantile value ≥535 μg/m^3^) and PM_2.5_ (the 90th-quantile value ≥304 μg/m^3^) led to an approximately 30% increase in daily CVD outpatient visits. The risk of an increase in CVD outpatient visits due to PM_10_ (≥500 μg/m^3^) and PM_2.5_ (≥300 μg/m^3^) reached 7.781 (CI: 1.681–36.024) and 8.72 (CI: 1.523–49.934), respectively, and their AR% reached 87.15 and 88.53%, respectively. Although the concentrations of PM_10_ and PM_2.5_ and the values of ORs (calculated by different concentrations of air pollutants) in our study were different from those in other studies (usually calculated by an OR per 10-μg/m^3^ increase), our results also revealed that PM_10_ and PM_2.5_ had a stronger impact on CVD outpatient visits, and the trend of the impact was similar to that in other studies.

### Impact of SO_2_ and NO_2_

The association of SO_2_ and NO_2_ with CVD was also the most intensively studied, similarly to PM_10_ and PM_2.5_ [3–7, 9, 11, 13, 17, 19–21, 24–27]. Hospitalization or death due to heart failure has been associated with increases in SO_2_ (2.36% per 10 ppb, CI: 1.35–3.38) and NO_2_ (1.70% per 10 ppb, CI: 1.25–2.16) concentrations [10]. These pollutants have been significantly associated with an increase in myocardial infarction risk (NO_2_: 1.011, CI: 1.006–1.016; SO_2_: 1.010, CI: 1.003–1.017) [11]. However, some studies have shown that SO_2_ made no independent contribution to admissions for ischaemic heart disease, and SO_2_ became insignificant after controlling for PM_10_ [5]. On warm days (> 25 °C), SO_2_ had no significant positive associations with congestive heart failure [24]. In our study, daily CVD outpatient visits for SO_2_ and NO_2_ were increased only in the higher concentration of the 90th-quantile group (SO_2_ ≥ 228 μg/m^3^, NO_2_ ≥ 109 μg/m^3^), and the increased percentages (SO_2_ = 17.68%, NO_2_ = 14.98%) were lower than those for PM_10_, PM_2.5_ and CO by approximately 50%. All the ORs for the increase in CVD outpatient visits were also insignificant, which revealed that the impact of SO_2_ and NO_2_ on CVD may be smaller than that of PM_10_, PM_2.5_ and CO.

### Impact of carbon monoxide

CO was the earliest-studied gaseous pollutant related to CVD because it can lead to the accumulation of carboxyhaemoglobin and can reduce the oxygen-carrying capacity of the blood [3–5, 9, 11, 13, 17, 19–22, 24, 25, 27–32]. High concentrations of CO have caused myocardial infarction [33], and low concentrations of CO have been associated with angina [34, 35]. Many studies have revealed that CO levels were positively associated with hospital admissions for congestive heart failure in the single-pollutant and multipollutant models [9]. There was a 3.52% (CI: 2.52–4.54) increase in hospitalizations or mortality due to heart failure per 1-ppm increment of CO [10]. CO was the strongest predictor of cardiovascular visits in multipollutant models [20]. In our study, higher CO in the 90th- quantile group (CO ≥ 4.3 mg/m^3^) led to a 29.34% increase in daily CVD outpatient visits; the risk of the increase in CVD outpatient visits due to CO (≥3 mg/m^3^) reached 5.808 (CI: 1.016–33.217), and its AR% reached 82.78%, which revealed that CO was also a stronger risk factor (similar to PM_10_ and PM_2.5_).

### Impact of ozone

O_3_ was the most controversial air pollutant related to CVD. Several studies have shown that O_3_ has a positive correlation with CVD [3, 4, 8, 9, 16, 26, 30, 35]. Short-term inhalation of fine particulate matter and O_3_ has caused acute conduit artery vasoconstriction [16]. Two-pollutant models have indicated that the impact of O_3_ has been significant in combination with another pollutant on warm days [26]. The OR and 95% CI estimated from general additive models for an interquartile range increase in O_3_ (20.5 ppb) was 1.010 (CI: 1.002–1.017) [30]. However, other studies have shown that O_3_ was not associated with CVD or was negatively correlated [5, 10, 11, 13, 18, 19, 21]. All the main air pollutants, with the exception of O_3_, were significantly associated with an increase in the risk of myocardial infarction; for O_3_, the relative risk (RR) was 1.003 (CI: 0.997–1.010) [8]. Hospitalization or death due to heart failure was not associated with increases in O_3_ concentrations (0.46% per 10 ppb, CI: − 0.10-1.02) [10]. The RR of admission for ischaemic heart disease and 95% CI for interquartile range increases in O_3_ were 1.010 (0.990–1.032) [5]. In some studies, the analyses for O_3_ were restricted to the warm period (May to October) [36]. In our study, O_3_ levels in 365 days were negatively associated with CVD outpatient visits. However, there was no longer a negative relationship in the 183 days of the warm period, although CVD outpatient visits did not increase substantially. One study from southern China showed that the impact of O_3_ on CVD mortality was stronger during high-exposure months (September to November) after adjustments were made for PM_10_ [37]; these findings differed from our results. It is well known that O_3_ is the main component of photochemical smog (85%) that is the product of NO_2_ and volatile organic compounds under strong sunlight. The reason for this negative correlation in our study may be that the O_3_ concentration in Shijiazhuang City was significant different between the winter (for example, O_3_ averaged 13.6 μg/m^3^, air temperature averaged − 4.2 °C in January) and the summer (O_3_ averaged 148.5 μg/m^3^, air temperature averaged 24 °C in Jun), and that the amount of CVD outpatient visits was opposite to the concentration of O_3_ (averaged 274 people in January, 249 people in June, without adjusting any factors). The impact of air temperature on CVD outpatient visits was significant, but that of O_3_ was not significant, and there was no significant interaction between both of them (not shown). The our results after adjusting for air temperature showed an actual relationship between O_3_ and CVD outpatient visits, which were similar to other studies [1, 5, 11, 13, 18, 19, 21, 36]. The reason for the difference between the north and the south in China may be that O_3_ level in winter of Guangzhou was still high (O_3_ averaged 50 μg/m^3^, air temperature > 10 °C in January) [38], but O_3_ level in winter of Shijiazhuang was very low (averaged 13.6 μg/m^3^, air temperature averaged − 4.2 °C in January). Therefore, it should be paid an attention to the difference between the O_3_ concentration and CVD outpatient visits in the warm and cold periods, especially when there is a negative correlation.

In our supplemental files, whether for the risk of increasing CVD outpatient visits per unit increase of air pollutant or for Pearson partial correlation coefficient between daily CVD outpatient visits and air pollutants, the trend of both results was consistent with the trend of the results from the main table and figures. The supplemental results also revealed that the impact of PM_10_, PM_2.5_ and CO on CVD outpatient visits was stronger than that of SO_2_, NO_2_ and O_3_.

Our study has three strengths. First, we explored the relationship between the different concentrations of air pollutants and the amount of CVD outpatient visits in a severe haze-fog city using GLM (LSmeans) and logistic regression (ORs). Second, our study revealed that when the concentration of air pollutants increased, an increase was seen not only in the number of CVD outpatient visits but also in the risk of the increase in CVD. Third, although the analytical methods differed from each other, the impacting trend of air pollutants in this study was consistent with those of other studies; specifically, PM_10_, PM_2.5_, and CO were the main risk factors for the increase in CVD outpatient visits. However, our results also have limitations. First, the climate in northern China (Beijing or Shijiazhuang) and southern China (Shanghai or Guangzhou) varies greatly, and our results for O_3_ may be different from those of other cities in southern China. Second, because Shijiazhuang is only one of many heavily polluted cities in northern China, our analytical method and results need to be validated in studies of other heavily polluted cities in the future.

## Conclusions

The present study explored the association between daily air pollutants and CVD outpatient visits in a severe haze-fog city in 2013 using GLM (LSmeans) and logistic regression (ORs). Our findings revealed that the elevated concentration of five air pollutants, with the exception of O_3_, increased the number of CVD outpatient visits and that PM_10_, PM_2.5_ and CO also heightened the risk of the increase in CVD outpatient visits. The trend for the impact of air pollutants on CVD outpatient visits was the same as those of other studies, although the analytical methods were changed in this study. The results of this study will provide a reference for early warnings for managers or physicians in the hospital and the staff of the Centers for Disease Control and Prevention before severe haze-fog occurs.

## Supplementary information

**Additional file 1: Figure S1** and **Tables S1-S3**. Table S2 and S3 provides the ORs of daily CVD outpatient visits in lag 0 per increase in different units of each pollutant, and the Pearson partial correlation coefficient between of them, respectively. These additional results also supported our conclusion.

## Data Availability

The datasets are available from the corresponding author on reasonable request.

## Abbreviations

AR%: Attributable risk percent.
BTH: Beijing-Tianjin-Hebei
CI: Confidence interval
CO: Carbon monoxide
CVD: Cardiovascular disease
LSmeans: Least squares mean
NO_2_: Nitrogen dioxide
O_3_: Ozone
ORs: Odds ratios
PM_10_ and PM_2.5_: Particulate matter
SO_2_: Sulphur dioxide
