# Supplementary figures and images for: Air pollutants and outpatient visits for cardiovascular disease in a severe haze-fog city: Shijiazhuang, China
Source: BMC Public Health. 2019 Oct 24;19:1366. doi: 10.1186/s12889-019-7690-4 (PMC6814061; doi:10.1186/s12889-019-7690-4)

**Supplemental Files**


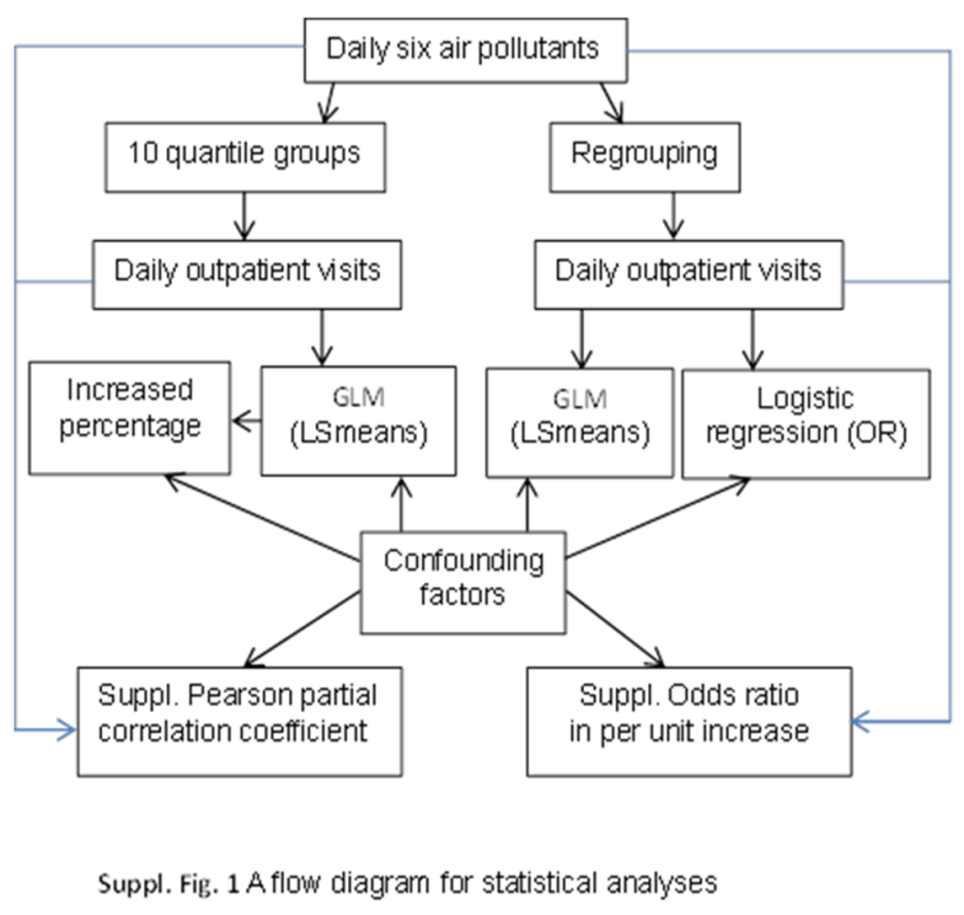

Supplement: Supplementary file 1 — Additional file 1: Figure S1 and Tables S1-S3. Table S2 and S3 provides the ORs of daily CVD outpatient visits in lag 0 per increase in different units of each pollutant, and the Pearson partial correlation coefficient between of them, respectively. These additional results also supported our conclusion. [file 12889_2019_7690_MOESM1_ESM.docx]
